# The pathologic and genomic evolution of primary malignant phyllodes tumors of the breast: retrospective cohort study and case-control genomic analysis

**DOI:** 10.1093/oncolo/oyaf012

**Published:** 2025-02-08

**Authors:** Carmine Valenza, Dario Trapani, Francesca Maria Porta, Edoardo Olmeda, Aurora Gaeta, Luca Boscolo Bielo, Federica Conversano, Tommaso Martino De Pas, Grazia Castellano, Celeste Santoro, Elena Battaiotto, Eltjona Mane, Sara Coppola, Fabio Conforti, Denise Mattar, Sara Gandini, Paolo Veronesi, Elena Guerini Rocco, Elisabetta Pennacchioli, Giuseppe Curigliano

**Affiliations:** Division of New Drugs and Early Drug Development for Innovative Therapies, European Institute of Oncology, IRCCS, Milan 20141, Italy; Department of Oncology and Hemato-Oncology, University of Milan, Milan 20122, Italy; Division of New Drugs and Early Drug Development for Innovative Therapies, European Institute of Oncology, IRCCS, Milan 20141, Italy; Department of Oncology and Hemato-Oncology, University of Milan, Milan 20122, Italy; Division of Pathology, European Institute of Oncology, IRCCS, Milan 20141, Italy; Department of Oncology and Hemato-Oncology, University of Milan, Milan 20122, Italy; Division of Pathology, European Institute of Oncology, IRCCS, Milan 20141, Italy; Department of Experimental Oncology, European Institute of Oncology, IRCCS, Milan 20141, Italy; Division of New Drugs and Early Drug Development for Innovative Therapies, European Institute of Oncology, IRCCS, Milan 20141, Italy; Department of Oncology and Hemato-Oncology, University of Milan, Milan 20122, Italy; Division of Pathology, European Institute of Oncology, IRCCS, Milan 20141, Italy; Medical Oncology Division, Cliniche Humanitas Gavazzeni, Bergamo 24125, Italy; Division of New Drugs and Early Drug Development for Innovative Therapies, European Institute of Oncology, IRCCS, Milan 20141, Italy; Department of Oncology and Hemato-Oncology, University of Milan, Milan 20122, Italy; Division of New Drugs and Early Drug Development for Innovative Therapies, European Institute of Oncology, IRCCS, Milan 20141, Italy; Department of Oncology and Hemato-Oncology, University of Milan, Milan 20122, Italy; Division of New Drugs and Early Drug Development for Innovative Therapies, European Institute of Oncology, IRCCS, Milan 20141, Italy; Department of Oncology and Hemato-Oncology, University of Milan, Milan 20122, Italy; Division of Pathology, European Institute of Oncology, IRCCS, Milan 20141, Italy; Division of Melanoma, Soft Tissue Sarcomas and Rare Tumors, European Institute of Oncology, IRCCS, Milan 20141, Italy; Medical Oncology Division, Cliniche Humanitas Gavazzeni, Bergamo 24125, Italy; Division of Breast Surgery, European Institute of Oncology, IRCCS, Milan 20141, Italy; Department of Experimental Oncology, European Institute of Oncology, IRCCS, Milan 20141, Italy; Department of Oncology and Hemato-Oncology, University of Milan, Milan 20122, Italy; Division of Breast Surgery, European Institute of Oncology, IRCCS, Milan 20141, Italy; Department of Oncology and Hemato-Oncology, University of Milan, Milan 20122, Italy; Division of Pathology, European Institute of Oncology, IRCCS, Milan 20141, Italy; Division of Melanoma, Soft Tissue Sarcomas and Rare Tumors, European Institute of Oncology, IRCCS, Milan 20141, Italy; Division of New Drugs and Early Drug Development for Innovative Therapies, European Institute of Oncology, IRCCS, Milan 20141, Italy; Department of Oncology and Hemato-Oncology, University of Milan, Milan 20122, Italy

**Keywords:** malignant phyllodes tumors of the breast, breast tumor, fibroadenoma, MED12

## Abstract

**Background:**

In patients with phyllodes tumors of the breast, the presence of *mediator of RNA polymerase II transcription subunit 12 homolog* mutations (*MED12*m) and a history of previous fibroadenoma may predict better outcomes. To aid in the prognostication of malignant phyllodes tumors of the breast (B-MPT), we assessed the prognostic value of fibroadenoma-like areas (supposed to have stemmed from a pre-existing fibroadenoma) and *MED12*m, in patients with resected primary B-MPTs.

**Methods:**

We conducted a single-center, retrospective, cohort study including all consecutive patients aged ≥18 years old, with non-metastatic B-MPT, who underwent surgery from January 2000 to December 2021. The endpoints were the cumulative incidences of all recurrences, according to the presence of fibroadenoma-like areas, reviewed by 3 reference pathologists. A nested, case-control genomic analysis was performed to evaluate the association between *MED12*m, the presence of fibroadenoma-like areas, and cancer recurrences.

**Results:**

Eight-nine patients were included, with 47% of tumors exhibiting Fibroadenoma-Like Areas (FLA+). These areas were not significantly associated with local recurrence (5-year cumulative incidence in FLA+ vs FLA-: 13.0, 95%CI [4.6-25.9] vs 23.6, 95%CI [11.9-37.5]; *P* = .14) or distant recurrence (5-year cumulative incidence in FLA+ vs FLA-: 12.5, 95%CI [4.5-25.0] vs 8.9, 95%CI [2.8-19.5], *P* = .61), at a median follow-up of 6.7 years. *MED12*m was not associated with distant recurrences or the presence of fibroadenoma-like areas.

**Conclusions:**

Half of B-MPTs are characterized by the presence of fibroadenoma-like areas. This pathologic feature is not significantly associated with lower distant and local recurrences nor with the presence of *MED12* mutations.

**Implication for practice**

Half of B-MPTs is characterized by the presence of fibroadenoma-like areas, supposed to have stemmed from a pre-existing fibroadenoma. This pathologic feature is not significantly associated with lower distant and local recurrences nor with the presence of *MED12* mutations, as previously suggested. Pending the demonstration of its clinical validity and utility, the comprehensive genomic profiling of B-MPTs should be performed only in the context of clinical studies, and not inform the clinical practice, at this stage.

## Introduction

Malignant phyllodes tumors of the breast (B-MPT) are rare fibroepithelial tumors, accounting for less than 1% of all breast neoplasms.^1^ Their treatment consists of wide local surgical excision with negative margins (≥1 cm). Still, a clear understanding of the prognosis of resected B-MPTs is missing, and the role of adjuvant systemic therapies is very debated.^2^

A third of patients with a primary B-MPT will eventually experience disease recurrence, with similar rates of local and distant recurrences, as recently reported.^2^ Because of such a tangible risk of recurrence, there is an urgent need to refine prognostication of this tumor in the clinical practice, as to inform adjuvant treatment decision-making. Current knowledge on adjuvant regimens, for example with doxorubicin and dacarbazine, suggests no impact on survival.^3^

The identification of prognostic biomarkers is instrumental to identifying the subgroup of patients potentially eligible for adjuvant therapies in risk-informed clinical trials. In this regard, the most important prognostic factor is tumor size; other suggested prognostic factors include: heterologous differentiation, multifocal disease, mitosis, grading, and stromal overgrowth.^2,4,5^

As far as emerging biomarkers are concerned, Abe et al. demonstrated that the history of previous fibroadenoma is associated with better overall survival in patients with primary B-MPT. Instead, Ng et al. showed that *mediator of RNA polymerase II transcription subunit 12 homolog* mutations (*MED12)*-mutant phyllodes of the breast are characterized by improved disease-free-survival rates.^6^ Combining these observations, Pareja et al. showed an association between the presence of fibroadenoma-like areas, in the context of a borderline/malignant phyllodes tumor of the breast, and the presence of *MED12* mutations.^7^ They further hypothesized that borderline/malignant phyllodes tumor could derive from a fibroadenoma/benign phyllodes tumor, through a *MED12*-dependent pathway, or could arise de novo through the acquisition of genetic alterations, that is, *EGFR* mutations or amplifications (*MED12*-indepentend pathway).

However, the prognostic role of fibroadenoma-like areas and *MED12* mutations in the context of primary B-MPT (ie, excluding patients with borderline and/or benign phyllodes tumors) is still unknown.

This study aims to evaluate the prevalence and the prognostic role of fibroadenoma-like areas and *MED12* mutations in patients with resected primary B-MPT.

## Methods

### Study population

We conducted a single-center, retrospective cohort study and an exploratory, nested, case-control genomic analysis at the European Institute of Oncology (IEO) IRCCS, Milan, Italy.

We collected data from consecutive patients with the following inclusion criteria: age 18 years or more at diagnosis, histologically confirmed diagnosis of B-MPT, primary (non-metastatic) disease at whole body computed tomography (CT) scan, availability of adequate tissue samples, surgery from January 2000 to December 2021. Patients with borderline MPT were excluded.

Two pathologists (FMP and EO) independently re-reviewed all histological sections from the primary B-MPT to assess the following pathologic features: heterologous differentiation, number of mitoses, stromal overgrowth, necrosis, giant cells, presence of fibroadenoma-like areas (defined as intracanalicular, pericanalicular, or myxoid areas of low stromal cellularity, lacking cytologic atypia, and mitotic activity).^7^ Discordant cases were reviewed together with a third pathologist (EGR) to reach a consensus.

All information was obtained through access to medical records. The research was conducted in accordance with the principles stated in the Declaration of Helsinki and with the principles of good clinical practice.

### Biomarker analysis

Eight patients with distant recurrence (cases) and 8 without distant recurrence (controls) were included in the exploratory, nested, case-control genomic analysis, and underwent comprehensive genomic profiling using the Oncomine Comprehensive Assay v3.^8^ Controls were pair-matched to cases based on the presence of fibroadenoma-like areas. This small sample of patients included in the biomarker analysis was due to resource constraints.

Fourteen unstained slides with 4-μm-thick sections from representative formalin-fixed, paraffin-embedded (FFPE) tissue blocks were used for nucleic acid extraction. Nucleic acids were extracted using seven slides with the Maxwell RSC DNA and RNA FFPE Kit (Promega, Madison) following the manufacturer’s instructions and then quantified with the Quantus Fluorometer (Promega).

For the OCA assay, libraries were prepared by using an Ion AmpliSeq DL8 kit (ThermoFisher Scientific) on an Ion Chef system (ThermoFisher Scientific) following manufacturer instructions. After library reamplification and barcoding, libraries were diluted at 30 pM and newly loaded into the Ion Chef instrument for automatic template preparation and loading chip. Finally, the barcoded chip was sequenced on an Ion S5 System (Thermo Fisher Scientific) following manufacturer instructions. The sequence data analysis was carried out by using customized analysis parameters on Torrent Suite 5.16.

### Endpoints

The main endpoints included the cumulative incidences of any recurrence (local or distant recurrence) defined as the radiological finding of metastatic or local recurrent disease, whichever occurs first; the cumulative incidence of distant recurrence, defined as the radiological finding of metastatic disease; the cumulative incidence of local recurrence, defined as the radiological finding of local recurrent disease.

The main endpoint of the exploratory genomic analysis was the odds ratio (OR) measuring the association between the presence of *MED12* mutations and distant recurrences.

### Statistical analyses

Descriptive statistics were used to analyze patients’ characteristics. Clinical and biological variables were grouped into standard categories whenever reasonable. Continuous variables were expressed as the median and interquartile interval (IQR) and were compared by the Wilcoxon rank-sum test. Categorical variables were expressed as numbers and proportions (%) and were compared by Fisher’s exact test or chi-square test, as appropriate.

The incidence of recurrence events was estimated with the competing-risk method and reported as a rate per person-years using cumulative incidence function (CIF).^9^ Death and any recurrence were considered as competing events. Similarly, local recurrence and distant recurrence were considered as competing events, and the first that occurred was considered as the relevant event.

Comparison of relative risk was assessed by taking into account competing risks, this allows formal assessment of differences in associations using the Gray test.^10^

Furthermore, to reduce the risk of confounding due to imbalance, a propensity score analysis (PS) was performed. The PS was estimated using a logistic regression model, in which the presence of fibroadenoma-like areas was regressed on the following covariates: size ≥5 cm, necrosis, stromal overgrowth, mitoses ≥15/10 HPF, heterologous differentiation, and multifocal disease.

Competing risk Cox regression models is used on the whole cohort to assess the role of fibroadenoma-like areas, adjusting by risk strata identified with the PS.

Fisher’s exact test and ORs were used in the exploratory nested case-control genomic analysis.

All tests are performed 2-sided at a significance level of *α* = 0.05. Statistical analyses are performed using R Studio software (version 4.2.2 2022–10–31).

## Results

A total of 89 eligible patients were included: their characteristics are reported in **Table 1**. All patients underwent radical surgery for B-MPT, 5 (6%) also received adjuvant anthracycline-based chemotherapy and 12 (13%) had additional adjuvant radiotherapy.

**Table 1.** Patient and tumor characteristics.

Characteristic	All patients (*N* = 89)
Age at diagnosis, median (IQR)	46 (40-57)
Tumor size ≥ 5 cm, *n* (%)	46 (52)
Multifocal disease, *n* (%)	5 (6)
Heterologous differentiation, *n* (%)	30 (34)
Mitosis, median number per 10 HPF (IQR)	11 (10-15)
Mitosis ≥ 15/10 HPF, *n* (%)	23 (26)
Stromal overgrowth, *n* (%)	58 (65)
Necrosis, *n* (%)	22 (25)
Giant cells, *n* (%)	24 (28)
Fibroadenoma like areas, *n* (%)	42 (47)
Adjuvant chemotherapy, *n* (%)	5 (6)
Adjuvant radiotherapy, *n* (%)	12 (14)

**Abbreviations:** HPF, high power field; IQR, interquartile range; *n*, number.

On the pathology report of primary surgery, the presence of fibroadenoma-like areas (FLA+) was described in 42 (47%) patients and was associated with lower mitoses (≥15/10 HPF in FLA + vs FLA-: 14% vs 36%; *P* = .02) (**Table 2**). At the independent revision of all histological sections, only 2 discordant cases emerged, which were discussed together with a third pathologist.

**Table 2.** Patient and tumor characteristics according to the presence of fibroadenoma-like areas.

Characteristic	FLA+ (*N* = 42)	FLA- (*N* = 47)	*P*-value^*^ (FLA + vs FLA-)
Age at diagnosis, median (IQR)	45 (42-58)	46 (39-54)	0.54
Tumor size ≥ 5 cm, *n* (%)	24 (57)	22 (47)	0.33
Multifocal disease, *n* (%)	3 (7)	2 (4)	0.66
Heterologous differentiation, *n* (%)	13 (31)	17 (36)	0.60
Mitosis, median number per 10 HPF (IQR)	11 (10-13)	11 (10-16)	0.39
Mitosis ≥ 15/10 HPF, *n* (%)	6 (14)	17 (36)	0.02
Stromal overgrowth, *n* (%)	27 (64)	31 (66)	0.87
Necrosis, *n* (%)	11 (26)	11 (23)	0.76
Giant cells, *n* (%)	11 (26)	13 (28)	0.99
Adjuvant chemotherapy, *n* (%)	1 (2)	4 (9)	0.37
Adjuvant radiotherapy, *n* (%)	6 (14)	6 (13)	0.83

^*^Wilcoxon rank sum exact test; Pearson’s Chi-squared test; Fisher’s exact test.

**Abbreviations**: FLA, fibroadenoma-like areas; HPF, high power field; IQR, interquartile range; *n*, number.

After a median follow-up of 6.7 (IQR: 4.35-10.33) years, 27 (30%) patients experienced a disease recurrence: 18 (20%) a loco-regional relapse and 14 (16%) a distant relapse.

**Figure 1.**
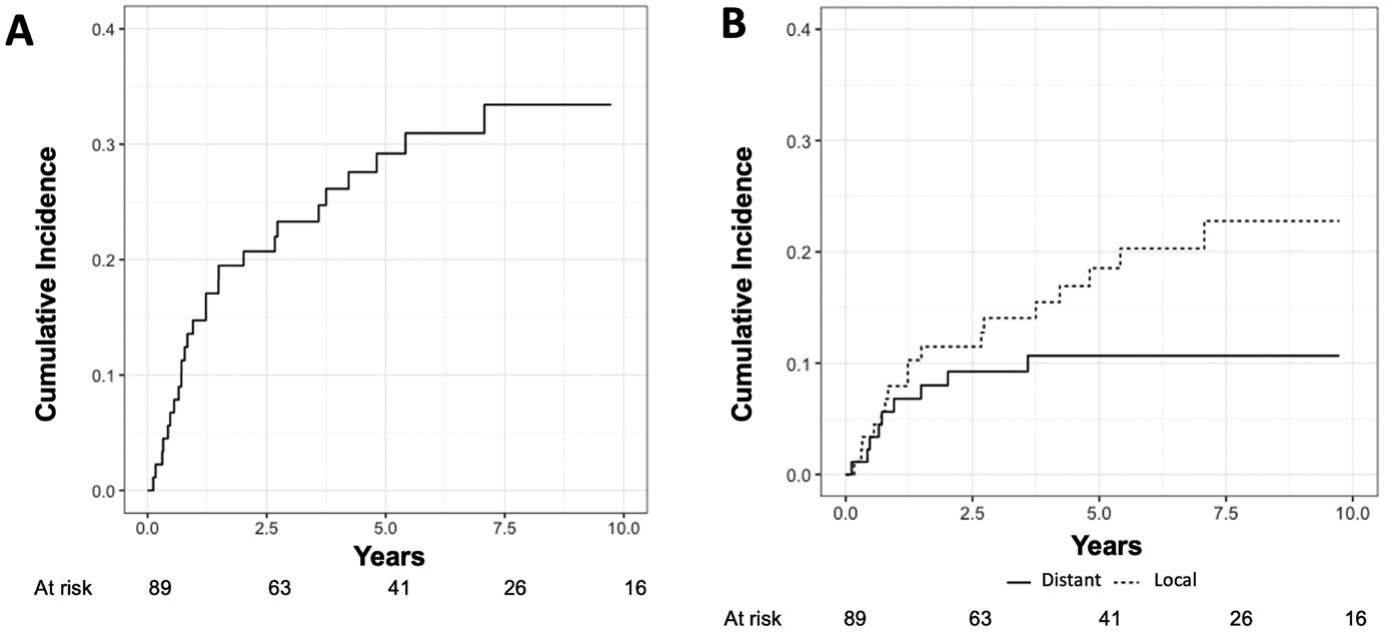
Competing risk analysis of cumulative incidence of any recurrence (A) and of distant and local recurrences (B).

The cumulative incidence at 5 years of any recurrence was 29.2% (95% confidence interval [CI]: 19.6-39.4) (Figure 1A), and of local and distant recurrences was 18.5% (95% CI: 10.8%-27.9%) and 10.7% (95% CI, 5.2%-18.4%), respectively (Figure 1B).

**Figure 2.**
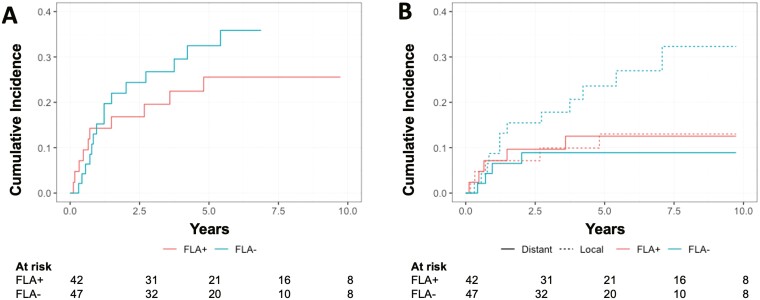
Competing risk analysis of cumulative incidence of any recurrence (A), and distant and local recurrences (B) according to the presence of fibroadenoma-like areas. **Abbreviation**: FLA, fibroadenoma-like areas.

At univariate analysis, the presence of fibroadenoma-like areas was not associated with any recurrence (5-year cumulative incidence in FLA+ vs FLA-: 25.6% [95% CI: 13.0%-40.2%] vs 32.5% [95% CI: 18.8%-46.9%]; *P* = .37), distant recurrence (5-year cumulative incidence in FLA+ vs FLA-: 12.5% [95% CI: 4.5%-25.0%] vs 8.9% [95% CI: 2.8%-19.5%]; *P* = .61) and local recurrence (5-year cumulative incidence in FLA+ vs FLA-: 13.0% [95% CI: 4.6%-25.9%] vs 23.6% [95% CI: 11.9%-37.5%]; *P* = .14) (Figure 2).

After adjusting for the propensity score, no significative effect was observed for the FLA status on any recurrence (*P*-value = .55), local recurrence (*P*-value = .20), and distant recurrence (*P*-value = .42).

Sixteen patients were included in the exploratory, nested, case-control, and genomic analysis (**Table 3**). Baseline characteristics were well balanced among cases and controls.

**Table 3.** Characteristics of patients included in the exploratory nested case-control genomic analysis.

Characteristic	Distant recurrence (*N* = 8)	No distant recurrence (*N* = 8)	*P*-value (cases vs controls)
Age at diagnosis, median (IQR)	52 (46-56)	58 (37-66)	.60
Tumor size ≥ 5 cm, *n* (%)	3 (38)	5 (63)	.62
Multifocal disease, *n* (%)	1 (13)	0 (0)	>.99
Heterologous differentiation, *n* (%)	2 (25)	4 (50)	.61
Mitosis ≥ 15/10 HPF, *n* (%)	4 (50)	2 (25)	.61
Necrosis, *n* (%)	4 (50)	3 (38)	>.99
Giant cells, *n* (%)	4 (50)	3 (38)	>.99
Fibroadenoma like areas, *n* (%)	4 (50)	4 (50)	>.99

**Abbreviations**: HPF, high power field; IQR, interquartile range; *n*, number.

**Figure 3.**
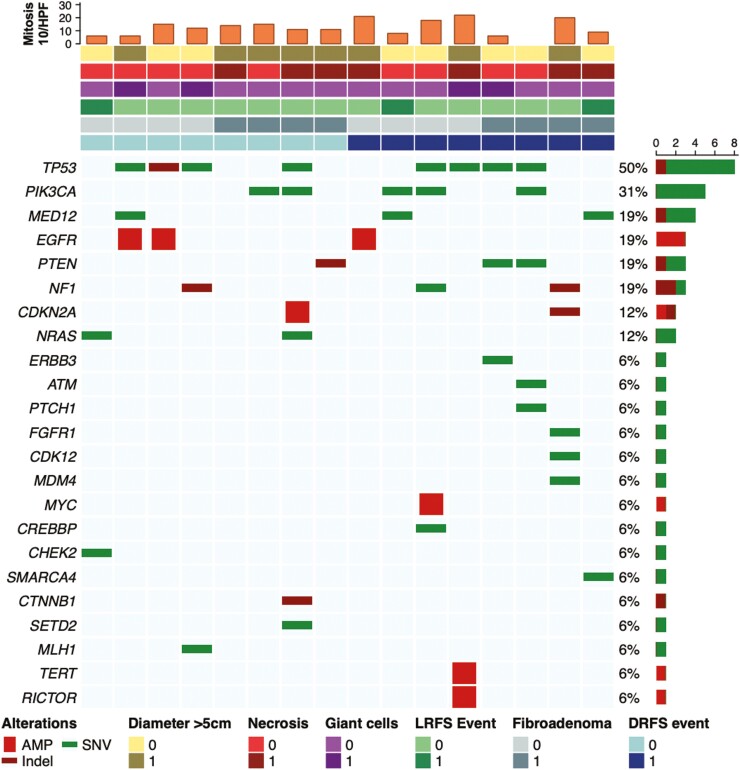
Genomic repertoire of malignant phyllodes tumors of the breast included in the genomic analysis.

Three B-MPTs harbored a *MED12* mutation, 2 among cases and 1 among controls (**Figure 3**). *MED12* mutations were not associated with distant recurrences (*MED12* mutations in cases vs controls: 2/8 vs 1/8; OR: 0.45; 95% CI: 0.01-10.79; *P*-value > .99) nor with the presence of fibroadenoma-like areas (*MED12* mutations in FLA+ vs FLA-: 1/8 vs 2/8; OR: 2.21; 95% CI: 0.09%-156.81; *P*-value > .99). *TP53* alterations and *EGFR* alterations were equally distributed among cases and control. No association emerged between other genomic alterations and the presence of fibroadenoma-like areas (Supplementary Table S1).

## Discussion

In this study, we reported that the presence of fibroadenoma-like areas in B-MPTs appears not to dissect the prognosis of patients with B-MPT nor to identify B-MPTs with specific genomic features. To the best of our knowledge, this is the largest study addressing specifically the issue of the pathologic and genomic evolution of B-MPT, excluding patients with benign and borderline tumors.

We demonstrated that the presence of fibroadenoma-like areas, as independently assessed by two pathologists, was observed in nearly a half of patients: these areas are usually interpreted as a cornerstone to consider that the cancer has stemmed from a pre-existing fibroadenoma, defining its pathogenetic route. Consistently, this pathologic feature was associated with a lower mitotic activity, as observed also by Pareja et al.^7^ in a study including both patients with borderline (*N* = 5) and malignant (*N* = 11) phyllodes tumor. However, in Pareja’s study, fibroadenoma-like areas were observed in all (5/5) borderline tumors but only in 18% (2/11) of malignant tumors, potentially suggesting a biological continuum between fibroadenomas and borderline phyllodes tumors, but not with the malignant ones.

We showed that the presence of fibroadenoma-like areas is not associated with better outcomes in patients with B-MPT. Conversely, in a study on 36 patients with B-MPT, Abe et al observed that B-MPT derived from fibroadenoma’s transformation have better outcomes than *de novo* B-MPTs.^4^ Among them, 11 (31%) patients had a previous history of fibroadenoma; in particular, 10 were detected with a B-MPT in the area near the scar of the previous breast surgery. These patients had a better overall survival (OS) compared to patients (*N*=25) with de novo B-MPT (median OS: not reached vs ~130 months; log-rank test: 0.055). However, this observation is limited by the small sample size, by the fact that the prior history of fibroadenoma was extracted from the patients’ medical record and not based on a pathological finding—which is less accurate than demonstrating a pathologic feature like fibroadenoma-like areas, and the likely closer follow-up of patients with previous fibroadenoma, which may be associated to earlier interception and faster management (ie, length time bias).

In our exploratory genomic analysis, *MED12* mutations were not associated with the presence of fibroadenoma-like areas. The mutations of this gene involved in the transcriptional regulation of gene expression have been observed in 65%-85% of benign and borderline phyllodes tumors and decrease in B-MPT (20-40% of cases), indicating their occurrence as an early event in the evolution of phyllodes tumors.^6,7,11,12^ Consistently, Pareja et al^7^ showed that *MED12* mutations were significantly more frequent in phyllodes tumors with fibroadenoma-like areas (71% vs 11%; *P* < .05), while *EGFR* alterations were significantly less frequent (14% vs 78%; *P* < .05). However, as stated, Pareja’s study included and pooled both patients with borderline and malignant tumors in the genomic analysis. Indeed, after restricting the observation only to patients with B-MPT, the association between *MED12* mutations and fibroadenoma-like areas becomes non-significant (1/2 vs 1/9; exact Fisher’s test; *P* = .32). Nevertheless, this evidence is exploratory and should be interpreted with caution, due to the small sample of patients with B-MPT included both in our (*N* = 16) and in Pareja’s (*N* = 11) biomarker analyses.

Lastly, we showed that *MED12* mutations were not associated with a reduced rate of distant recurrences. Conversely, Ng et el^6^ demonstrated that *MED12*-mutant phyllodes tumors yield a longer disease-free survival (DFS) compared to *MED12* wild-type ones (adjusted hazard ratio: 9.99; 95%CI, 1.55-64.42); however, 89% (86/97) of patients included in the study had a benign or borderline tumor and the DFS by *MED12* status in patients with B-MPT (*N* = 11) was not disclosed. However, due to the small number of DFS events in this subgroup (2/11), the benefit associated with *MED12* mutations was likely driven by patients with benign and borderline tumors.

However, many clinically actionable mutations and biomarkers for immunotherapy response have been identified in patients with B-MPT. In particular, a study on 135 patients with B-MPT showed that 21.4% of them were PD-L1-positive via Dako 22C3 assay (CPS ≥ 1) and that several tumors harbored genomic alterations with approved indications in other tumor types, including pathogenic *PIK3CA*, *EGFR* Exon 19/20 insertions, *KRAS* G12C (*N* = 1), *FGFR* fusions (*N* = 1), *BRAF* V600E mutations (*N* = 4), and *BRCA2* (N = 1).^13^

The limitations of our analysis include the retrospective observational design of the study that, however, included all consecutive patients; the sampling of specimens included in the study, which was performed for clinical purposes and not tailored for the assessment of the presence of fibroadenoma-like areas; the small number of patients included in the genomic analysis.

## Conclusion

A half of B-MPTs is characterized by the presence of fibroadenoma-like areas, potentially supposed to have stemmed from a pre-existing fibroadenoma. This pathologic feature is not significantly associated with lower distant and local recurrences nor with the presence of *MED12* mutations.

A deeper dissection of pathologic and genomic characteristics of B-MPTs is needed, in multicenter, prospective studies, to identify the subgroup of patients at higher risk of recurrence, potentially deserving adjuvant systemic treatments. Pending this data, the comprehensive genomic profiling of B-MPTs has not demonstrated clinical validity and utility and should be performed in the context of clinical studies.

## Supplementary Material

oyaf012_suppl_Supplementary_Tables_1

## Data Availability

The data underlying this article will be shared on reasonable request to the corresponding author.
