# Supplementary material for: The pathologic and genomic evolution of primary malignant phyllodes tumors of the breast: retrospective cohort study and case-control genomic analysis
Source: Oncologist. 2025 Feb 8;30(2):oyaf012. doi: 10.1093/oncolo/oyaf012 (PMC11806198; doi:10.1093/oncolo/oyaf012)
Supplement: oyaf012_suppl_Supplementary_Tables_1 [file oyaf012_suppl_supplementary_tables_1.docx]

**Supplementary table 1.** Univariate analysis of the association between genomic alterations and the presence of fibroadenoma-like areas.

| Odd ratio | p-value | Adjusted  p-value | Lower limit of confidence interval | Higher limit of confidence interval | **Gene** | n  (controls) | n  (cases) | %  (controls) | %  (cases) |
| --- | --- | --- | --- | --- | --- | --- | --- | --- | --- |
| Inf | 0.20 | 1 | 0.45 | Inf | **EGFR** | 3/8 | 0/8 | 0.375 | 0.000 |
| 2.60 | 0.62 | 1 | 0.26 | 32.60 | **TP53** | 5/8 | 3/8 | 0.625 | 0.375 |
| 0.00 | 1.00 | 1 | 0.00 | 39.00 | **ERBB3** | 0/8 | 1/8 | 0.000 | 0.125 |
| 0.00 | 0.20 | 1 | 0.00 | 2.21 | **PTEN** | 0/8 | 3/8 | 0.000 | 0.375 |
| 0.00 | 1.00 | 1 | 0.00 | 39.00 | **ATM** | 0/8 | 1/8 | 0.000 | 0.125 |
| 0.00 | 1.00 | 1 | 0.00 | 39.00 | **PTCH1** | 0/8 | 1/8 | 0.000 | 0.125 |
| 0.58 | 1.00 | 1 | 0.03 | 7.36 | **PIK3CA** | 2/8 | 3/8 | 0.250 | 0.375 |
| 0.00 | 1.00 | 1 | 0.00 | 39.00 | **FGFR1** | 0/8 | 1/8 | 0.000 | 0.125 |
| 2.21 | 1.00 | 1 | 0.09 | 156.81 | **NF1** | 2/8 | 1/8 | 0.250 | 0.125 |
| 0.00 | 0.47 | 1 | 0.00 | 5.21 | **CDKN2A** | 0/8 | 2/8 | 0.000 | 0.250 |
| 0.00 | 1.00 | 1 | 0.00 | 39.00 | **CDK12** | 0/8 | 1/8 | 0.000 | 0.125 |
| 0.00 | 1.00 | 1 | 0.00 | 39.00 | **MDM4** | 0/8 | 1/8 | 0.000 | 0.125 |
| 2.21 | 1.00 | 1 | 0.09 | 156.81 | **MED12** | 2/8 | 1/8 | 0.250 | 0.125 |
| Inf | 1.00 | 1 | 0.03 | Inf | **MYC** | 1/8 | 0/8 | 0.125 | 0.000 |
| Inf | 1.00 | 1 | 0.03 | Inf | **CREBBP** | 1/8 | 0/8 | 0.125 | 0.000 |
| 1.00 | 1.00 | 1 | 0.01 | 89.51 | **NRAS** | 1/8 | 1/8 | 0.125 | 0.125 |
| Inf | 1.00 | 1 | 0.03 | Inf | **CHEK2** | 1/8 | 0/8 | 0.125 | 0.000 |
| 0.00 | 1.00 | 1 | 0.00 | 39.00 | **SMARCA4** | 0/8 | 1/8 | 0.000 | 0.125 |
| 0.00 | 1.00 | 1 | 0.00 | 39.00 | **CTNNB1** | 0/8 | 1/8 | 0.000 | 0.125 |
| 0.00 | 1.00 | 1 | 0.00 | 39.00 | **SETD2** | 0/8 | 1/8 | 0.000 | 0.125 |
| Inf | 1.00 | 1 | 0.03 | Inf | **MLH1** | 1/8 | 0/8 | 0.125 | 0.000 |
| Inf | 1.00 | 1 | 0.03 | Inf | **TERT** | 1/8 | 0/8 | 0.125 | 0.000 |
| Inf | 1.00 | 1 | 0.03 | Inf | **RICTOR** | 1/8 | 018 | 0.125 | 0.000 |
